# Tissue engineering and future directions in regenerative medicine for knee cartilage repair: a comprehensive review

**DOI:** 10.3325/cmj.2024.65.268

**Published:** 2024-06

**Authors:** Dragan Primorac, Vilim Molnar, Dimitrios Tsoukas, Ilona Uzieliene, Carlo Tremolada, Petar Brlek, Emil Klarić, Dinko Vidović, Marija Zekušić, Jolita Pachaleva, Eiva Bernotiene, Adrian Wilson, Ali Mobasheri

**Affiliations:** 1 Center for Genetics and Personalized Medicine, St. Catherine Specialty Hospital, Zagreb, Croatia; 2 School of Medicine, Josip Juraj Strossmayer University of Osijek, Osijek, Croatia; 3 Medical School, University of Split, Split, Croatia; 4 Forensic Science Program, Department of Biochemistry & Molecular Biology, The Pennsylvania State University, State College, PA, USA; 5 The Henry C. Lee College of Criminal Justice and Forensic Sciences, University of New Haven, West Haven, CT, USA; 6 REGIOMED Kliniken, Coburg, Germany; 7 Medical School, University of Rijeka, Rijeka, Croatia; 8 Faculty of Dental Medicine and Health, Josip Juraj Strossmayer University of Osijek, Osijek, Croatia; 9 Medical School, University of Mostar, Mostar, Bosnia and Herzegovina; 10 National Forensic Sciences University, Gujarat, India; 11 Clinic for Orthopaedic Surgery, St. Catherine Specialty Hospital, Zagreb, Croatia; 12 Orthopaedic Clinic for Advanced Arthroscopic Sports and Regenerative Medicine, MITERA Hospital, Athens, Greece; 13 Department of Regenerative Medicine, State Research Institute Centre for Innovative Medicine, Vilnius, Lithuania; 14 Image Regenerative Clinic, Milan, Italy; 15 Department of Traumatology, Sestre Milosrdnice University Hospital Center, Zagreb, Croatia; 16 School of Medicine, University of Zagreb, Zagreb, Croatia; 17 School of Dental Medicine, University of Zagreb, Zagreb, Croatia; 18 Department of Transfusion and Regenerative Medicine, Sestre Milosrdnice University Hospital Center, Zagreb, Croatia; 19 The Regenerative Clinic, London, UK; 20 Research Unit of Health Sciences and Technology, Physics and Technology, Faculty of Medicine, University of Oulu, Oulu, Finland; 21 Department of Regenerative Medicine, State Research Institute Centre for Innovative Medicine, Vilnius, Lithuania; 22 Department of Joint Surgery, First Affiliated Hospital of Sun Yat-sen University, Guangzhou, Guangdong, China; 23 World Health Organization Collaborating Center for Public Health Aspects of Musculoskeletal Health and Aging, Université de Liège, Liège, Belgium

## Abstract

This review evaluates the current landscape and future directions of regenerative medicine for knee cartilage repair, with a particular focus on tissue engineering strategies. In this context, scaffold-based approaches have emerged as promising solutions for cartilage regeneration. Synthetic scaffolds, while offering superior mechanical properties, often lack the biological cues necessary for effective tissue integration. Natural scaffolds, though biocompatible and biodegradable, frequently suffer from inadequate mechanical strength. Hybrid scaffolds, combining elements of both synthetic and natural materials, present a balanced approach, enhancing both mechanical support and biological functionality. Advances in decellularized extracellular matrix scaffolds have shown potential in promoting cell infiltration and integration with native tissues. Additionally, bioprinting technologies have enabled the creation of complex, bioactive scaffolds that closely mimic the zonal organization of native cartilage, providing an optimal environment for cell growth and differentiation. The review also explores the potential of gene therapy and gene editing techniques, including CRISPR-Cas9, to enhance cartilage repair by targeting specific genetic pathways involved in tissue regeneration. The integration of these advanced therapies with tissue engineering approaches holds promise for developing personalized and durable treatments for knee cartilage injuries and osteoarthritis. In conclusion, this review underscores the importance of continued multidisciplinary collaboration to advance these innovative therapies from bench to bedside and improve outcomes for patients with knee cartilage damage.

Regenerative medicine holds significant promise for knee cartilage repair and prevention of osteoarthritis (OA), due to its ability to harness the body's natural healing processes to restore damaged joint tissues (1-3). Traumatic knee injuries, particularly those that affect the articular cartilage component, are associated with an increased risk of OA (4). Healthy articular cartilage tissue is avascular, alymphatic, aneural, and is characterized by limited self-repairing capacity; thus the restoration or promoting endogenous repair of articular cartilage represents a significant clinical challenge (5).

Traditional and conservative treatment options for OA, such as physical therapy, weight management, and pain management using pharmacological agents have limited potential for long-term clinical management of OA (6,7). Surgical interventions like microfracture and arthroplasty may provide symptomatic relief but often fail to restore the structure and function of the native articular cartilage (8,9). Therefore, regenerative approaches aim to address this crucial limitation of cartilage repair by promoting the regeneration of functional cartilage tissue (10,11). Mesenchymal stem cell (MSC) therapy has been available for some time as a promising approach for the treatment of OA, especially in the knee. From its establishment, MSC therapy has yielded encouraging clinical outcomes, including pain relief, functional recovery, and improvement in radiological findings, evidenced by increased concentration of glycosaminoglycans in cartilage measured by delayed gadolinium-enhanced MRI of cartilage (12-15). Despite promising results, the introduction of MSC therapy in the guidelines of leading professional societies is hindered by the lack of sufficiently large and well-designed studies, as well as the heterogeneity of the method itself, including significant variations in MSC acquisition systems (16).

At the same time, tissue engineering approaches combine cells, scaffolds, and signaling molecules to create functional tissue constructs for cartilage repair (17,18). Tissue-engineered cartilage constructs aim to mimic the composition, structure, and mechanical properties of native cartilage while promoting host integration and remodeling (19,20). Strategies such as cell-based tissue engineering, where cells are cultured on scaffolds *in vitro* before implantation, and acellular tissue engineering, where scaffolds are pre-seeded with growth factors or bioactive molecules, have shown promise in preclinical studies of cartilage repair (20). Advances in biomaterials, bioprinting technologies, and tissue culture techniques continue to drive innovation in tissue engineering for cartilage repair.

Here, we provide a comprehensive overview of tissue engineering approaches, highlighting future directions and research priorities in the field of regenerative medicine for the treatment of knee OA. By examining current methodologies and emerging innovations, we hope to underscore the potential and challenges of developing effective regenerative therapies for this prevalent and debilitating condition.

## Tissue engineering approaches for knee cartilage repair

**Figure 1:**
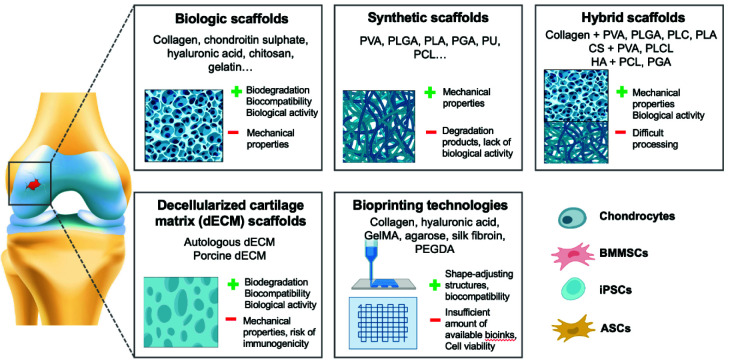
Pros and cons of different tissue engineering approaches for knee cartilage repair.

Scaffolds play a pivotal role in cartilage tissue engineering, serving as the foundation for supporting structures, creating an ideal micromechanical environment, and delivering the essential biochemical signals required for cell growth and chondrocyte differentiation. Tissue engineering approaches for cartilage repair incorporate a variety of strategies, each with its own set of benefits and challenges (Figure 1).

Scaffolds provide a three-dimensional framework for cell attachment, proliferation, and matrix deposition, facilitating the regeneration of functional cartilage tissue. Various natural and synthetic biomaterials, such as collagen, gelatin, hyaluronic acid, and polycaprolactone, have been used as scaffolds for cartilage repair (21-26). These scaffolds can be seeded with cells (eg, MSCs, chondrocytes) or growth factors to enhance tissue regeneration. Scaffold-based approaches for regenerative medicine offer structural support, controlled release of bioactive molecules, modulation of the microenvironment, and zonal cartilage engineering (27,28). There is also an ongoing interest in developing bioinspired nanofibers for cartilage repair (29). However, despite all these efforts, challenges remain in designing scaffolds with optimal biomechanical properties, biocompatibility, and degradation kinetics to promote integration with native tissue and long-term stability in the clinical setting (30). Scaffold-based approaches can be divided into biologic scaffolds, synthetic scaffolds, hybrid scaffolds, decellularized cartilage matrix (dECM) scaffolds, and bioprinting technologies.

## Injectable natural scaffolds for cartilage regeneration with a special emphasis on microfragmented adipose tissue

In the domain of tissue engineering, there is a growing interest in injectable scaffolds designed for the repair or regeneration of cartilage. Such scaffolds are developed to support the regrowth of cartilage through a biocompatible, biodegradable, and well-hydrated three-dimensional framework that emulates the natural extracellular matrix (ECM) of cartilage (31). Essential characteristics of an optimal injectable scaffold are straightforward injectability, superior biocompatibility, the capability to replicate the properties of cartilaginous ECM, and the potential to seamlessly merge with the existing cartilage tissue (31,32).

Recent advancements in injectable hydrogels have shown promise in cartilage tissue engineering. These hydrogels offer properties such as good biocompatibility, adaptability to irregular cartilage defect surfaces, and strong plasticity (33). They are designed to fill defect areas inside joints smoothly, while not integrating into the surrounding healthy tissue (31). Additionally, injectable hydrogels can encapsulate cells and deliver bioactive molecules efficiently through stimuli-responsive release mechanisms (31).

Various materials and fabrication approaches have been explored to enhance the mechanical properties of scaffolds and to improve their integration with the surrounding cartilage (31). The use of nanocomposites and advanced formulations like microspheres and nanoparticles has been investigated for controlled drug delivery within these scaffolds (32). Furthermore, injectable hydrogels have been developed to present biochemical cues in a controllable manner to promote cartilage regeneration (33).

Injectable scaffolds for cartilage repair or regeneration surely represent a promising strategy in tissue engineering. They offer a versatile approach to delivering cells, growth factors, and bioactive molecules efficiently to aid in the regeneration of articular cartilage. Ongoing research focuses on enhancing the structural properties, cross-linking techniques, and controlled release strategies of injectable scaffolds.

Despite promising ongoing research, the translational value remains complex. Some of the key challenges that need to be addressed include (32,33) the following:

• Long duration of culture: Traditional cell-hydrogel constructs used in injectable cartilage regeneration often require extended culturing, which can delay the treatment process and limit their clinical translation;

• Infection risk: Cell-hydrogel constructs have been associated with a high probability of infection, posing a significant concern for patient safety and successful outcomes;

• Poor cartilage formation capacity: A critical challenge is the limited ability of cell-hydrogel constructs to form robust and functional cartilage tissue, impacting their effectiveness in promoting cartilage regeneration;

• Structural compactness: Injectable hydrogels, such as gelatin methacrylate (GelMA), may face challenges due to their compact structure, which can affect their ability to mimic the natural extracellular matrix (ECM) of cartilage effectively;

• Biocompatibility and integration: Ensuring excellent biocompatibility and seamless integration with surrounding native cartilage tissue remains a challenge;

• Controlled release mechanisms: Achieving precise control over the release of bioactive molecules within injectable hydrogels is crucial for promoting optimal cartilage regeneration outcomes.

**Figure 2:**
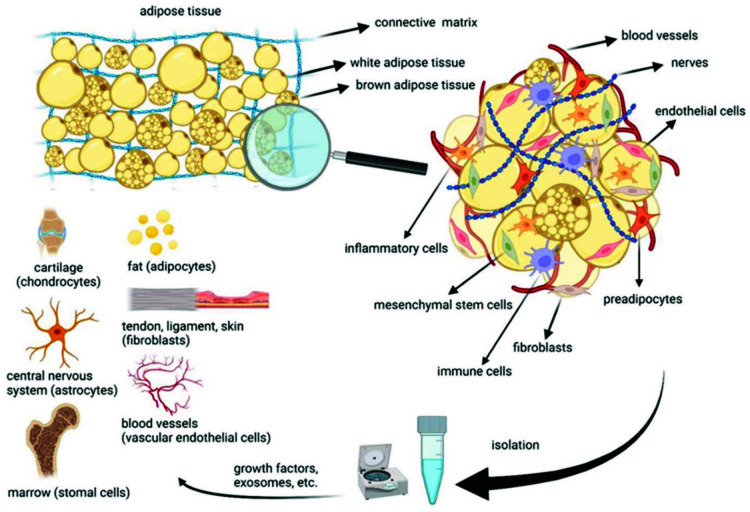
Adipose tissue composition and the isolation process for regenerative medical applications. The diagram illustrates the heterogeneous cell population within adipose tissue, including white and brown adipocytes, mesenchymal stem cells (MSC), preadipocytes, fibroblasts, endothelial cells, and immune cells, all embedded in a connective matrix with associated blood vessels and nerves. The process of isolating these components for therapeutic use is depicted. The MSCs that could differentiate into chondrocytes, fibroblasts, adipocytes, stromal cells, endothelial cells, astrocytes, etc, are highlighted.

Most of these challenges have been potentially solved in the last decade by an innovative and alternative approach that employs a biological autologous scaffold, microfragmented adipose tissue (MFAT), obtained by a patented device developed by Tremolada et al (34,35). This device has been extensively used clinically in many thousands of patients worldwide for different indications, showing remarkable safety and effectiveness. Adipose tissue components and the cell isolation process are depicted in Figure 2.

The innovative concept of a living injectable scaffold was first performed *in vitro* in 2016, when it was demonstrated that an intra-articular injection of MFAT can act as a one-step repair strategy for cartilage defect. It enabled the outgrowth of cells that repopulate fragments of damaged cartilage or even another synthetic or biological devitalized scaffold from a tissue bank (36). This research indicated that when lipoaspirate is treated for three-weeks with chondrogenic growth factors in a floating culture system, its composition transforms significantly. The tissue gradually loses fat content, while connective tissue, characterized by abundance of glycosaminoglycans (GAG) and collagen type I, becomes denser. This change enhances the tissue’s structural integrity. The described encouraging laboratory findings indicate that using lipoaspirate as an injectable, autologous, biologically active scaffold within a joint might lead to several beneficial outcomes: 1) it could transform into a fibrous tissue that underpins the mechanical load of deteriorated cartilage; 2) it might stimulate the surrounding chondrocytes to multiply and synthesize the ECM; and 3) it could supply cells directly to the injury site, potentially aiding in the regeneration or restoration of damaged or absent cartilage.

In 2019, independent research groups demonstrated *in vivo* the use of Lipogems® MFAT as a scaffold for cartilage defect repair (37-39). Further elaborate research and characterization efforts demonstrated both *in vivo* and *in vitro* that Lipogems® MFAT (and even devitalized MFAT) was a highly effective natural scaffold for drug delivery with potential applications in many cancer chemotherapies or other therapies that require a local long-term action or a lower systemic toxicity (40). This method has already been used clinically in veterinary medicine and is going to be approved as a compassionate therapy in human trials (41). Even better drug delivery systems are now in development using exosomes derived by Lipogems® MFAT (42).

Therefore, MFAT derived from the Lipogems® device has emerged as a promising natural scaffold for cartilage repair due to its autologous nature, regenerative properties, and ability to provide a conducive environment for cartilage healing compared to other traditional methods or scaffolds. The main advantage of MFAT as a scaffold is that it is also an implantable natural living tissue graft that stays in place for a long time. Compared to other scaffolds or bioreactors, MFAT offers advantages such as safety and maintaining the secretion of active cytokines for a very extended period, aiding in repairing damaged areas and reducing inflammatory responses. Moreover, it acts as the perfect natural scaffold for the pericytes present on the surface of the intact micro-clusters, which over time produces MSCs that can function most naturally. The method shows improved *in vitro* and *in vivo* results, as well as more reliable radiological and clinical results in cartilage regeneration, even in advanced cases, than SVF and cultured expanded MSCs (12,38,43,44).

## Synthetic scaffolds

Synthetic polymers are being more and more recognized as promising scaffold materials for cartilage repair because of their ability to be processed easily and their capacity to be modified to achieve desired properties. Furthermore, synthetic materials used for producing scaffolds are more mechanically resistant and durable, as compared to the natural compounds (45). Frequently applied synthetic polymers are polyurethane (PU), poly(ethylene glycol) (PEG), polylactic acid (PLA), poly(ϵ-caprolactone) (PCL), and poly(lactic-co-glycolic acid) (PLGA) (46).

PU has several desirable properties, including low cytotoxicity, high oxygen permeability, and high biocompatibility, which make it a suitable material for various biomedical applications. Additionally, PU is biodegradable and displays mechanical characteristics similar to natural materials (47). PU scaffolds with suitable mechanical properties can facilitate the development of a functional cartilage-like ECM. The mechanical properties and degradation of PU are influenced by the methods used for its synthesis and scaffold processing. Specifically, scaffolds fabricated using freeze-drying techniques and PU synthesized via a water-based process promote chondrogenic differentiation of MSCs (48). Furthermore, PU scaffolds promoted better growth of chondrocytes and increased matrix secretion than PLA scaffolds (48). According to mechanical properties, the use of PU is widespread in repairing meniscal defects (49,50).

PLA is a biodegradable and biocompatible polyester produced by the fermentation of carbohydrates and is degraded to lactic acid (51). Furthermore, PLA scaffolds better promote the proliferation of chondrocytes than PLGA and PU scaffolds (48,52). The application of PLA for scaffolding has certain limitations, particularly in relation to low cell adhesion on the surface.

PGA is a hydrophobic polymer with mechanical properties suitable for cell adhesion. However, PGA alone, as a chondroconductive graft, was insufficient to stimulate cartilage healing. PGA scaffolds, in conjunction with autologous bone marrow concentrate, have stimulated the production of ECM and functioned as a carrier for the implantation of MSCs into rabbit model cartilage defects (53). Furthermore, PGA scaffolds stimulated chondrogenic differentiation of human chondrocytes, with increased collagen II and aggrecan and decreased collagen I gene expression (54). However, the main disadvantages of PGA scaffolds are their acidic degradation products and rapid degradation (55).

PCL is widely recognized for its cost-effectiveness, stability, and mechanical strength, although it is not hydrophilic. It is a frequently utilized biodegradable polyester in medical applications and demonstrates a more gradual degradation rate compared to other polyesters. PCL porous scaffolds efficiently promoted cell proliferation and chondrogenesis of MSCs (56). The use of nanostructured porous PCL scaffolds in a rabbit model successfully restored articular cartilage defects. PCL scaffolds exhibited good surface regularity and strong structural integrity, in addition to promoting the proliferation of chondrocytes (56).

PLGA scaffolds, made up of both PGA and PLA, are known for their controlled biodegradability and low immunogenicity (57). PLA and PLGA have been confirmed by the United States Food and Drug Administration for clinical use, and they are promising materials for various medical and pharmaceutical applications owing to their ability to produce safe and non-toxic degradation products (58). PLGA offers appropriate degradation rates but suffers from poor mechanical properties. The use of a combination of PCL and PLGA polymers at various ratios enables the production of scaffolds that possess the necessary features for facilitating cell adhesion, proliferation, and differentiation. Scaffolds made from PCL and PLGA at a ratio of 80:20 successfully repaired all cartilage defects in rats (57).

Despite their advantages, many synthetic scaffolds have limitations, such as the uncontrolled degradation and release of acidic products (46), as the polylactic and polyglycolic acids are eventually broken down to lactic and glycolic acids. Although the degradation products are easily processed, they create an acidic environment that can negatively affect both the environment and cell behavior (59). In summary, although synthetic scaffolds exhibit superior mechanical properties compared to their natural counterparts, they cannot completely replicate the intricate structure of the ECM of articular cartilage and are limited in terms of cell adhesion and material degradation. Nevertheless, if these properties are enhanced and supplemented, synthetic scaffolds can serve as a promising tool for cartilage defect repairs.

## Hybrid scaffolds

Natural materials such as collagen, hyaluronic acid, and chondroitin sulfate show good biodegradation and biocompatibility properties, but their use alone for scaffolds is limited because of their poor mechanical properties (60). Synthetic materials lack desirable biological properties and cannot mimic cartilage ECM without modification. Hybrid scaffolds, which include features of both synthetic and natural materials, provide the necessary mechanical properties, biofunctionality, and tunable degradation for cartilage regeneration.

*Collagen-based scaffolds*. To enhance the mechanical properties of collagen scaffolds, they can be functionalized with various polymers, such as polyvinyl alcohol (PVA) and hydroxyapatite (61). However, the use of collagen with PLGA, PLC, and PLA is limited, as the processing of these polymers requires organic solvents, which denature collagen (61). The mentioned polymers could be combined with collagen as separate constructs. As an example, porous hybrid scaffolds with controlled pore size, prepared by hybridization of PLGA mesh and collagen I sponge, had excellent mechanical properties. PLGA-collagen scaffolds facilitated homogenous bovine articular chondrocyte distribution and increased aggrecan gene expression (62). To fabricate artificial articular cartilage that replicates the superficial and transitional zones, collagen-PVA nanofibers were electrospun onto a freeze-dried collagen sponge. The created scaffold mimicked the structure of cartilage surface and possessed good tensile strength (63).

*Chondroitin sulfate-based scaffolds*. Cartilage contains a physiological component known as chondroitin sulfate (CS), which offers numerous advantages, such as anti-inflammatory properties, the ability to absorb water and nutrients, and the potential to promote the growth of new cartilage cells. These qualities make CS an effective tool for repairing the structure and function of the articular cartilage (64). CS shows an anionic nature, which facilitates the binding of water by aggrecan and creates osmotic resistance to articular cartilage during compressive forces (61). The incorporation of PVA and CS via crosslinking produced mechanically stable scaffolds, which closely resembled the structure and composition of cartilage ECM, effectively addressing articular cartilage defects in a rat model (65). Hybrid scaffolds comprised of CS, PLCL, and silk fibroin promote the proliferation of chondrocytes and enhance the development of more mature cartilage-like tissues in defected rabbit articular cartilage *in vivo* (64).

*Hyaluronic acid-based scaffolds*. Hyaluronic acid is known to regulate cell functional properties, inflammation, and promotes cartilage regeneration. However, HA-based constructs have low degradation rates and poor mechanical properties, which limit the application of HA in cartilage tissue engineering (66). A combination of HA with PCL demonstrated mechanical properties that closely resembled those of human articular cartilage and preserved chondrocyte phenotype (67). Chondrotissue® scaffold (BioTissue AG, Zürich, Switzerland), composed of PGA and HA, is currently under clinical trial. Arthroscopically applied PGA-HA scaffolds immersed in platelet-rich plasma stimulated with bone marrow resulted in an improved repair of hyaline-like cartilage tissue. Obtained results reveal its potential to be used for hyaline cartilage regeneration (68).

To summarize, in the area of cartilage tissue engineering, significant progress has been made with the development of hybrid scaffolds that effectively incorporate the ideal characteristics of natural and synthetic materials. Natural materials such as collagen, hyaluronic acid, and chondroitin sulfate mimic the ECM of cartilage and offer excellent biodegradation and biocompatibility; however, their mechanical properties are limited. Furthermore, synthetic materials lack the biological properties that are essential for cartilage regeneration. Hybrid scaffolds can overcome the limitations of natural and synthetic materials by integrating the benefits of both types of materials, providing essential mechanical strength, attachment, biofunctionality, and controlled degradation. Therefore, hybrid scaffolds show great potential for use in cartilage tissue engineering.

## Decellularized cartilage matrix scaffolds

The intercellular communication is paramount to maintaining ECM homeostasis in articular cartilage. Additionally, this communication serves as the primary signaling pathway that regulates cartilage development (68). Current scaffolds, whether natural or synthetic, can only partially recreate the complex composition and arrangement of the ECM components.

Decellularized cartilage extracellular matrix (dECM) scaffolds are three-dimensional structures composed of cartilage tissue that have been processed to eliminate cellular components while keeping the original ECM. Decellularized scaffolds are used for the repair of knee cartilage defects by promoting cell infiltration and ECM production, which leads to integration with the surrounding tissues (69). The application of autologous dECM scaffolds is limited by the availability of donors and potential health risks associated with their use, particularly when treating significant cartilage defects. As a result, current research efforts have concentrated on the preparation of allogeneic and xenogeneic tissues for cartilage repair (70).

### Decellularization

Articular cartilage decellularization involves the use of detergents to dissolve the cartilage tissue and break down chondrocytes, resulting in the elimination of cellular debris and genetic material. Decellularization typically employs a combination of physical, chemical, and biological methods because the dense nature of cartilage can interfere with the full penetration of detergents. Physical methods include thawing with liquid nitrogen, forming ice crystals, and damaging the cells. Chemical methods (SDS and Triton X-100) disrupt interactions between proteins and lipids, whereas enzymes diminish DNA content by eliminating nuclear debris (71). To achieve optimal cartilage decellularization, the protocol should effectively eliminate all cellular components of the cartilage while preserving the structure and properties of the ECM (71). Different techniques can be applied to increase the porosity of dECM scaffolds. The use of laser-machined micropores enhanced both the formation and decellularization of the cartilage matrix without impacting its mechanical strength. In a rabbit model, the recellularization of scaffolds with autologous chondrocytes effectively repaired cartilage defects (70).

### Recellularization

To promote the chondrogenic properties and regeneration of dECM scaffolds, it is possible to recellularize them by using various cell types. dECM impacts SC differentiation by establishing a specific niche. Recellularization and cartilage regeneration are frequently performed by BMSCs because they can be easily obtained and have a high proliferation and differentiation potential (71). Additionally, synovium- and adipose-derived MSCs can also be used, although these cell types exhibited lower chondrogenic potential than BMSCs. Other MSC types are also employed. For example, recellularization of sheep dECM scaffolds with endometrium MSCs has demonstrated remarkable chondrogenic differentiation, as indicated by the significant upregulation of aggrecan and type II collagen (72). Chondrocytes in dECM also demonstrated increased GAG production but with lower differentiation capacity as compared with MSCs. Furthermore, the use of autologous chondrocytes is limited as it causes additional damage to the donor tissue (73).

### Functionalization of dECM with other materials

Decellularization influences the mechanical features of the cartilage ECM. In addition, the functionalization of dECM scaffolds with other materials enhances their mechanical properties. For instance, modification of the dECM with graphene oxide significantly improved the scaffold mechanical strength and enhanced cell adhesion, proliferation, and chondrogenesis *in vitro* (74).

Decellularized cartilage scaffolds combined with chemically cross-linked chitosan demonstrated enhanced biodegradation, swelling ratio, mechanical properties, biocompatibility, and chondrocyte attachment compared with pure chitosan scaffolds (75). In another study, dECM in combination with chitosan-protected cartilage, diminished knee joint pain in rats, and significantly delayed the progression of knee OA. dECM scaffolds are used together with PLGA microspheres to regulate the release of kartogenin. The scaffold complex prolonged the activity of kartogenin and promoted attachment, proliferation, and differentiation of BMSCs *in vitro*. Additionally, dECM/PLGA scaffolds with kartogenin resulted in the formation of superior hyaline-like neocartilage repair, which effectively integrated with the surrounding cartilage in a rabbit model (76).

Overall, the combination of decellularized cartilage scaffolds with advanced recellularization techniques and functionalization using various materials is a promising approach for repairing articular cartilage defects.

## Bioprinting technologies

3D bioprinting is another promising avenue for cartilage tissue regeneration. It allows precise deposition of biomaterials and creates structures that mimic cartilage matrix. Bioprinting enables the recreation of complex cartilage architectures, including the zonal organization crucial for optimal functionality. Bioprinted constructs provide a conducive environment for cell growth and, most importantly, cell differentiation. However, challenges still remain with mimicking the functional properties of cartilage and choosing the right bioink.

Bioinks in 3D bioprinting are usually heterogeneous, containing living cells or hydrogels/biomaterials, and are used according to the biological architecture of cartilage and other joint components. Materials used for developing cartilage repair bioinks are collagens, hyaluronic acid, silk fibroin, chitosan, GelMA, poly(ethylene glycol)-diacrylate (PEGDA), and agarose-based hydrogels (77). GelMA is the most frequently used and tested hydrogel for bioprinting. Numerous studies have shown its suitability and biocompatibility with cells. GelMA promoted cellular properties, such as migration and proliferation, as well as chondrogenic differentiation of MSCs by upregulating microRNA-410 in rabbits (78). It was also used in the development of Biopen, a bioprinter which manually deposits living cells embedded into biomaterials. Biopen bioink is a combination of GelMA with hyaluronic acid-methacrylate. They are crosslinked by UV light during bioprinting, which enables direct biofabrication of 3D-shaped constructs in damaged areas. Cells remained viable after seven days of such bioprinting, and the ability to directly control bioprintable material holds a huge promise in future surgeries (79).

Bioprinting is used for both regeneration of cartilage ECM and *in vitro* disease modeling. Disease modeling faces several limitations due to the integration of additional tissues by layering, vascularization, recreating mechanical properties of the construct, however, bioinks alone cannot recreate osteochondral units. Therefore, a combination of different hydrogels and scaffolds is being studied for developing mini-joints using bioprinters, or organ-on-chip platforms for disease modeling (80,81). For instance, GelMA was used together with oxidized methacrylated alginate (OMA) and PRG4 (lubricin)-transduced chondrocytes for the recreation of the cartilage surface zone, which is enriched with lubricin-producing chondrocytes. The developed bioink, containing 2% OMA and 14% GelMA, is the optimal formulation for lubricin secretion over 22 days in culture and is promising for the surface cartilage layer (82). Other studies have produced efficient osteochondral constructs by combining two hydrogels: one consisting of GelMA with nanohydroxyapatite for bone tissue and the other consisting of tyramine-hyaluronic acid for cartilage tissue. Osteoblasts and micropellet chondrocytes were encapsulated into two hydrogels and, after seven days of culture, the formation of osteochondral tissue was successfully confirmed by histological evaluation and RT-PCR (83). Another study showed encouraging results with bioink combining sodium alginate (SA), gelatin (GA), and hydroxyapatite (HA). SA-GA-HA scaffolds have demonstrated mechanical stability and biocompatibility with ATCD-5 cells and may provide beneficial clinical outcomes for cartilage tissue regeneration (84).

Some bioinks often incorporate a combination of growth factors, cells, and biomaterials, fostering an environment-stimulated chondrogenesis. For instance, to modulate BMP, TGFβ, and interleukin-1 (IL-1) signaling cascades in BMMSC-encapsulated silk fibroin gelatin (SF-G) bioinks, small molecules, such as LDN193189, TGFβ3, and IL1 receptor antagonist (IL1Ra), were conjugated to SF-G biomaterial to ensure sustained release, printability, and increased bioavailability. These bioprinted constructs, together with MSCs, are a novel strategy to produce cartilage constructs resistant to OA traits (85).

Despite notable strides, challenges persist, including ensuring the long-term viability of bioprinted constructs and achieving seamless integration with the host tissue. Ongoing research aims to refine bioprinting techniques, enhance cell viability, and advance post-implantation integration strategies for the prospect of personalized, precision-engineered solutions for OA.

## Future directions and research priorities in regenerative medicine for knee cartilage repair

### Development of advanced scaffolds and biomaterials

**Figure 3:**
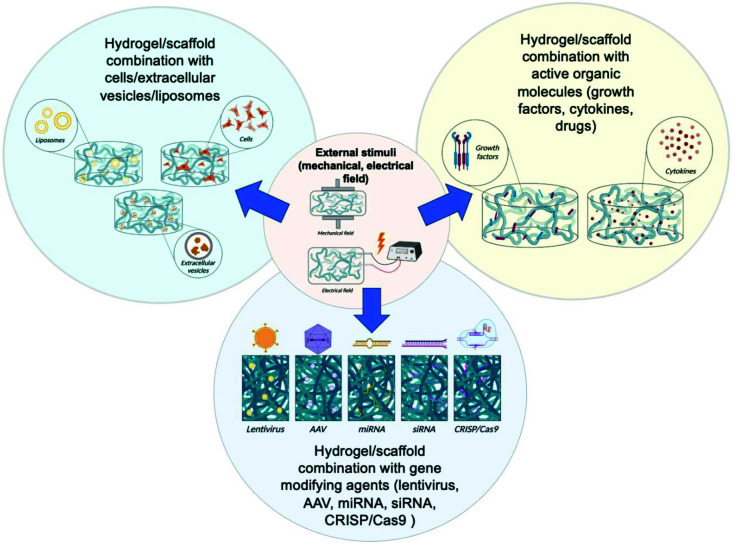
Future advanced scaffolds and biomaterials for cartilage tissue repair.

Key focus in the current research are innovative biomaterials prepared for therapeutic delivery. Advanced biomaterials should be designed to closely replicate the intricacies of healthy cartilage, from mechanical strength to biochemical composition, promoting seamless integration and functional recovery. Advanced biomaterials include loading with bioactive agents, such as growth factors/drugs, cells/extracellular vesicles/liposomes, or delivering specific genes, optimizing the regenerative process, and addressing the dynamic and mechanical needs of cartilage repair over time (Figure 3). An additional favorable aspect of advanced biomaterials should be their injectability, overcoming demanding implantation procedures.

One of the developing fields is the application of external, electrical or mechanical, stimuli for activation of scaffolds and cells. Activation of hydrogel seeded with chondrocytes by direct or indirect electrical stimulation was shown to be beneficial for cartilage tissue formation through activation of ion channels and extracellular matrix formation (86,87). Interestingly, one study proposed a computational model based on current research results for cartilage-tissue engineering in combination with electrical stimulation (88). It was demonstrated that a biodegradable, piezoelectric hydrogel, made of short electrospun poly-L-lactic acid nanofibers embedded inside a collagen matrix, can be injected into the joints and self-produce localized electrical cues under ultrasound activation to drive cartilage healing (89). A similar study has shown that biodegradable, piezoelectric poly(L-lactic acid) nanofiber scaffolds inserted into rabbit joints could generate electrical signals under applied force or joint load to promote chondrogenesis and cartilage regeneration (90). More piezoelectric scaffolds for cartilage, bone, and osteochondral tissue regeneration are discussed in a comprehensive review by Barbosa et al, and multiphasic scaffold use was detailed in a preclinical study by Chen et al (91,92).

Loading scaffolds/hydrogels with bioactive molecules, such as growth factors or drugs, is also an evolving and highly promising method for stimulating chondrogenesis and attenuating OA. A combination of GelMA with alanyl-glutamine has been proposed for *in vivo* metabolic activation of chondrocytes by sustained release of glutamine due to degradation of the construct. A study on chondrocytes demonstrated effective increase in mitochondrial membrane potential, while the intracellular content of reactive oxygen species was decreased, promoting damaged tissue repair (93). The release of active molecules is usually achieved by stimuli-responsive scaffolds. As an example, a hydrogel loaded with tannic acid and kartogenin is proposed as a cell-free scaffold for *in vivo* cartilage regeneration, which possesses ultra-durable mechanical properties and stage-dependent drug release behavior. The hydrogel could withstand 28 000 loading-unloading mechanical cycles while exhibiting fast shape memory at body temperature, anti-inflammatory properties, and the potential for minimally invasive surgery (94).

Gene editing tools, such as miRNA, siRNA, and CRISPR-Cas9, have also been widely studied in cartilage tissue repair. Usually, these molecules are being transferred using viral (adenovirus, lentivirus) or non-viral (liposomes, exosomes) methods, as well as in their naked form (95,96). Despite gene editions, cell-based biomaterials have gained most of the attention due to their direct contact with damaged tissue and stimulating its regeneration. Chondrocytes and MSCs are the most studied cell types for knee cartilage repair, while MSCs gained more attention due to their ease of access in the minimally invasive manner, in contrast to the isolation of chondrocytes, and personalized approach (97). Therefore, future research will embrace personalized approaches tailoring scaffolds and biomaterials to individual patient profiles. Factors such as age, activity level, and the extent of cartilage damage will guide the customization of regenerative solutions, ensuring precision in treatment outcomes.

These advancements promise not only the restoration of structural integrity in knee joints but also the development of personalized and effective solutions that can significantly improve the quality of life for individuals with knee cartilage issues.

### Advancements in gene therapy and gene editing

Gene therapy holds promise for cartilage repair by targeting the underlying mechanisms involved in cartilage degradation and promoting tissue regeneration (98-101). The rationale behind gene therapy for cartilage repair lies in addressing the fundamental factors contributing to cartilage degradation and impaired repair mechanisms. By delivering therapeutic genes directly into the affected joint, gene therapy aims to enhance chondrogenesis (cartilage formation), inhibit cartilage degradation, modulate inflammation, and promote tissue regeneration (102). Gene therapy offers the potential for sustained and localized expression of targeted genes, providing long-term benefits for cartilage repair (98-100).

Therapeutic targets in gene therapy strategies are diverse and include growth factors, ECM proteins, and anti-inflammatory factors. As outlined earlier, genes encoding growth factors such as *TGF-β*, *IGF-1*, *FGF*, and bone morphogenetic proteins (BMPs) can stimulate chondrogenic differentiation of MSCs and promote cartilage matrix synthesis (103-105). Genes encoding cartilage-specific ECM proteins such as type II collagen, aggrecan, and cartilage oligomeric matrix protein are ideal starting points for developing gene therapy strategies because they can enhance cartilage matrix production and promote tissue structural integrity (100). Anti-inflammatory factors associated with genes encoding anti-inflammatory cytokines such as interleukin-10 (IL-10) and interleukin-1 receptor antagonist (IL-1Ra) can modulate inflammation within the joint and mitigate cartilage damage especially if combined with growth factors (106-110). Inhibitors of matrix metalloproteinases (MMPs), enzymes involved in cartilage degradation, may also be considered for gene therapy strategies (111,112). Genes encoding inhibitors of MMPs can attenuate MMP activity and preserve cartilage integrity (113). Genes encoding chemokines or growth factors involved in stem cell recruitment can recruit endogenous stem cells to the site of cartilage injury, facilitating tissue repair.

Gene therapy involves the transfer of specific genes into target cells to stimulate tissue regeneration, enhancing the production of extracellular matrix and facilitating the differentiation of cells. It utilizes both viral and non-viral vectors for nucleic acid delivery, either directly into tissues (*in vivo*) or via transduced cells (*ex vivo*) (114). In preclinical studies, gene therapy has shown capacity in managing cartilage injuries and OA. Non-viral transgene delivery approaches include lipid nanoparticles, exosomes, cationic polymers, inorganic nanoparticles, and polymer hydrogels. Non-viral gene delivery technologies hold the potential for advancing disease-modifying therapeutics for OA and cartilage injuries.

Many studies have confirmed the feasibility of transferring various growth factors (IGF-1, FGF-2, and TGF-β) via lipid-based vectors into the chondrocytes to promote repair. However, this type of delivery is less efficient than the delivery via viral vectors (96,115,116). Viral vectors, including adenoviruses and lentiviruses, are also used for gene transfer, offering efficient targeted delivery into mammalian cells. Some studies have explored intra-articular injections of recombinant adeno-associated viruses to modulate cartilage metabolism and promote repair. Additionally, gene therapy has been employed to express therapeutic proteins, such as various growth factors and transcription factors, to enhance cartilage repair (115,117). CRISPR-based gene editing presents a promising avenue for developing new treatments for cartilaginous injuries and OA. For instance, adeno-associated viruses expressing CRISPR/Cas9 components have been used to target genes associated with cartilage degradation, reducing pain and supporting joint structure maintenance in animal models of OA (96). Both viral and non-viral gene delivery systems are used for treating articular cartilage defects, either through direct injection into the joint cavity or *ex vivo* manipulation of cells before delivery. *Ex vivo* gene delivery offers advantages in efficiency and safety compared to direct injection, particularly in terms of avoiding direct exposure to viral vectors. Stem-cell therapies, particularly those using MSCs, show potential for treating focal cartilage lesions by promoting chondrogenic differentiation (118). Combining gene therapy with tissue engineering holds promise for advancing cartilage repair strategies. Despite progress, further research is needed to optimize gene delivery methods and enhance the longevity of gene expression for effective treatment of cartilaginous injuries and OA.

### Cell therapy platforms

Various other cell therapy platforms are being developed for the treatment of OA through immunometabolic reprogramming of the inflammatory microenvironment of the synovial joint and the promotion of cartilage repair (119). The development of mammalian protein production platforms using virally transfected and irradiated protein packaging cell lines has offered the opportunity to develop “cellular factories” for the over-production of therapeutic proteins and growth factors, particularly in the context of developing intra-articular regenerative therapies such as TissueGene-C (120-122). In addition, PLX-PAD cells offer an off-the-shelf, placental-derived, mesenchymal stromal cell-like cell therapy for OA and potentially also for promoting muscle function for improved joint function (123-125). Clinical studies are currently being conducted to evaluate the efficacy of TissueGene-C in phase-III trials and PLX-PAD cells in phase-II trials for cartilage repair and OA treatment. Also, genetically modified anti-inflammatory macrophages may enter the arena for the promotion of cartilage repair (31).

### Development of personalized regenerative medicine strategies

Although various OA treatment options have been developed, currently no single choice of therapy has been shown to completely stop or delay OA progression (126). As OA has become recognized as a multi-faceted disease with different subtypes, a personalized approach to OA treatment seems the ultimate unmet need. Several OA drugs targeting different aspects of the disease and different OA pheno-endotypes have been used with diverse outcomes. OA presentations in individual patients are not restricted to one endotype and could change throughout the course of the disease, potentially even occurring as an overlapping endotype (such as inflammation and pain) (126). Therefore, monitoring the course of the disease and response to therapies help in the selection of appropriate treatment regimens. Therefore, recent studies have attempted to find biomarkers of different subgroups, and specific molecular endotypes of OA for the application of targeted treatment methods that could be precisely adapted to a specific patient's needs (127). The biomarker clustering analysis was suggested to stratify patients with OA into molecular endotypes based on 16 well-defined biochemical markers, which reflect different molecular pathways and ongoing pathophysiological processes (128). That study discovered three distinct OA phenotypes associated with the clusters (molecular endotypes): C1 – a low tissue turnover phenotype, C2 – a structural damage phenotype, and C3 – systemic inflammation phenotype. Such an approach could drive OA clinical trial stratification. It could also serve as the basis for the development of clear personalized phenotype-directed protocols for disease-modifying osteoarthritis drug (DMOAD) trials, which enable us to target subgroups with uniform OA characteristics,.

Several clinical trials have shown positive effects of MSC-based OA treatments on symptoms and joint function. However, these trials relied on small sample sizes, which greatly limits their capability to conclude on differences between MSCs of autologous and allogeneic origin and efficacy of treatment based on patient stratification to endotypes and phenotypes as well as MSC source (129). Further analysis of immunomodulatory and regenerative properties of different MSC products in correlation with patient profiling based on soluble biomarkers, imaging, and omics data are needed to provide a personalized selection of MSC therapies (129).

With the advancement of diagnostics based on extracellular vesicles (EVs) in multiple clinical fields, their application to individualize the treatment regimen for each OA patient could include not only a role as novel potential EV-based OA biomarkers, but also by, for example, assembling EVs with targeted therapeutic agents for achieving greatest effectiveness (130).

Biofabrication of scaffolds for cartilage tissue engineering and bioprinting of hydrogel constructs for biomimetic implants has already progressed to such a point that it is possible to mimic native cartilage architecture in various aspects, including gradient composition and zonal distribution (131). 3D design software could be used alongside imaging techniques, such as magnetic resonance imaging and computed tomography, to create precise multilayer structures to manufacture an implant to perfectly cover the defect size and shape (132).

Big-data tools such as proteomics, genomics, and metabolomics provide the potential to uncover more detailed OA profiles and novel therapeutic targets. Altered lipid metabolism in OA synovial fluid has been shown, therefore further metabolic profile studies, especially in association with genomic data, could be used to gain insight into OA subtypes and corresponding targets for disease-modifying drugs and interventional treatments (133). Multi-omics data sets of molecular and regulatory networks from diverse detection technologies, combined with individual patients’ clinical and sociodemographic data, hold promise for identifying unique patient endophenotypes, which can advance the application of personalized therapeutic strategies (134). Therefore, the personalization of OA treatment continues to be an ultimate goal in OA management.

### Translational research and clinical trial strategies

In the OA drug development pipeline, several DMOAD candidates are undergoing clinical trials, including drugs targeting anabolic activity, those inhibiting catabolic processes, and those targeting inflammation, pain, and metabolic syndrome pathways (126,135,136). For example, sprifermin, a growth factor, was shown to safely and effectively improve morphological parameters in knee OA (137). Similarly, although previously dismissed as ineffective, inhibitors of pro-inflammatory cytokine IL-1 such as canakinumab (investigated by a clinical trial primarily under indication of preventing cardiovascular events) have shown a surprising positive effect on the incidence rates of total hip and total knee replacement when compared to placebo (138). However, the implementation of novel therapies and precision medicine faces translational challenges to implementing laboratory discoveries into clinical practice.

Despite multiple clinical trials, only a few cell-based medicinal products have provided satisfactory results for the approval of the treatment of OA so far. For example, CARTISTEM, an umbilical cord blood-derived MSC product for knee OA, has been shown to be effective and safe after 7 years of follow-up (139). Another cell-based product, INVOSSA, a mixture of human allogeneic chondrocytes and cells engineered to overexpress TGF-β1, has been approved for clinical use (140). Both of these products have gained marketing approval in South Korea; however, the approval of INVOSSA was retracted and remains under appeal. Nonetheless, both products are currently undergoing clinical trials in the US.

EVs could become a great innovation in OA treatment. Immunomodulatory and regenerative effects of the parent cells are still maintained in EVs, while their clinical application is subject to fewer regulatory obstacles as they are considered a cell-free product. The storage, distribution, and administration of EVs to patients is easier, as opposed to the relevant cell therapies, which are often complicated by requirements for cell storage, short survival after transplantation, and difficulty in directing cell migration (141). However, further advancements in EV characterization and isolation at an industrial scale still have to be developed (130).

A crucial feature of the development of novel engineered cartilage tissue constructs is the biofabrication of scaffolds that provide structural support for cellular transplants. Improvements in scaffold manufacturing, such as novel artificial and biomimetic materials, bioprinting techniques, and inclusion of bioactive factors, have advanced cartilage tissue engineering; however, their clinical application possibilities are still limited (142). Several considerations should be explored, including biocompatibility of the applied materials, integration of the construct into native tissues, long-term stability of graft morphology, and bioprinting parameters that sustain cell viability and timely biodegradation of the scaffold (142).

Gene therapy has been extensively considered in the treatment of OA; however, several challenges obstruct the possibility of the current implementation of such treatment strategies. Viral gene transfer systems have been widely used, but due to their cost, complicated manufacturing and storage, as well as immunogenic properties, other, novel non-viral alternatives (such as liposomal and lipid-based systems, polymers, and nucleic-acid conjugates) have been proposed (96). Nevertheless, due to the suboptimal efficacy of these non-viral gene delivery systems demonstrated so far, they need to be further optimized for translational application in clinical trials.

Osteoarthritis Research Society International has developed recommendations for designing, conducting, and reporting of clinical trials that focus on modifying symptoms or structures in individuals with knee OA. These recommendations include randomization of patients, stratification of subphenotypes of OA, blinding procedures, study design, and methods of drug administration to prevent disclosure of assignment to participants and study staff (143,144).

Sensitive biomarkers or their combinations that provide insights into disease progression and responses to treatment, in association with clinical and imaging data, are pivotal in the design of OA clinical trials (145).

## Conclusion

Regenerative medicine holds immense promise for revolutionizing the treatment of knee cartilage damage by offering novel strategies to promote tissue regeneration, restore joint function, and alleviate symptoms associated with cartilage injuries and OA (146). While significant progress has been made in preclinical and early clinical studies of cartilage repair, further research is needed to optimize regenerative approaches, validate their safety and efficacy in larger patient populations, and translate them into routine clinical practice (147-150). Continued research and development are crucial to addressing the existing challenges and realizing the full potential of regenerative medicine for improving the lives of individuals affected by cartilage damage. Further progress in this area will depend on improvements in clinical trial design and increased collaboration between researchers, clinicians, engineers, and industry partners through public-private partnerships to accelerate the development, clinical validation, and adoption of regenerative therapies for knee cartilage repair (3,151-153).
